# Preventive or promotive effects of *PRNP* polymorphic heterozygosity on the onset of prion disease

**DOI:** 10.1016/j.heliyon.2023.e13974

**Published:** 2023-02-24

**Authors:** Hideaki Kai, Kenta Teruya, Atsuko Takeuchi, Yoshikazu Nakamura, Hidehiro Mizusawa, Masahito Yamada, Tetsuyuki Kitamoto

**Affiliations:** a Department of Neurological Science, Tohoku University Graduate School of Medicine, Sendai, Miyagi 980–8575, Japan; b Department of Neurochemistry, Tohoku University Graduate School of Medicine, Sendai, Miyagi 980-8575, Japan; c Division of Public Health, Center for Community Medicine, Jichi Medical University, Shimotsuke, Tochigi 329-0498, Japan; d Department of Neurology, National Center of Neurology and Psychiatry, Kodaira, Tokyo 187-0031, Japan; e Division of Neurology, Department of Internal Medicine, Kudanzaka Hospital, Chiyoda-ku, Tokyo 102-0074, Japan

**Keywords:** Creutzfeldt-jakob disease, Prion, *PRNP*, Genetic prion disease, Polymorphism, Electrostatic interaction, Dimer, Codon 129, Codon 219, Heterozygosity, CJD, Creutzfeldt-Jakob disease, E, glutamate, GSS, Gerstmann-Straussler-Scheinker disease, K, lysine, M, methionine, OR, odds ratio, PrP, prion protein, V, valine

## Abstract

The polymorphic heterozygosity of *PRNP* at codon 129 or 219 prevents the onset of sporadic Creutzfeldt-Jakob disease (sCJD). We investigated the association between polymorphic genotypes at codon 129 or 219 and comprehensive prion disease onset using non-CJD as a reference. EK heterozygotes at codon 219, versus EE homozygotes, showed a preventive effect on the extensive prion diseases―sCJD, genetic CJD (gCJD) with V180I or M232R mutation, and Gerstmann-Straussler-Scheinker disease with P102L mutation. No preventive effect was observed for E200K-gCJD and dura-grafted CJD (dCJD) in 129 MV and 219 EK heterozygotes. It was suggested that unlike other prion diseases, E200K-gCJD may not benefit from the preventive effect of 219 EK heterozygosity because complementary electrostatic interactions between PrP molecules at K200 and E219 might make homodimer formation easier. Comparison of sCJD and dCJD indicates that 219 EK heterozygosity strongly inhibits de novo synthesis of PrP^Sc^ (initial PrP^Sc^ formation), but does not inhibit accelerated propagation of existing PrP^Sc^.

## 1 Introduction

Human prion diseases are rare and fatal neurologic disorders, the causes of which are sporadic, genetic, or acquired by infection. Sporadic Creutzfeldt-Jakob disease (sCJD) is the most common human prion disease. All the prion diseases feature the accumulation of pathological aggregates (PrP^Sc^) of the cellular prion protein (PrP^C^) in the central nervous system [1].

The *PRNP* gene encodes PrP^C^ and genetic prion diseases are exclusively caused by its mutations. It is generally supposed that *PRNP* pathogenic mutations in genetic prion diseases lower the threshold for structural change from PrP^C^ to PrP^Sc^ and promote the generation of PrP^Sc^ molecules [2]. A common polymorphism of *PRNP* at codon 129 is either methionine (M) or valine (V) [3]. This polymorphism affects the characteristics of PrP molecules and the clinicopathology and vulnerability in prion diseases [1,[4], [5], [6], [7], [8], [9], [10], [11], [12], [13], [14]]. The 129 MV heterozygosity confers prevention of the onset of sCJD and the promotion of developing genetic CJD (gCJD) with the V180I mutation [6,13,15,16]. Another polymorphism of *PRNP* at codon 219 is either glutamate (E) or lysine (K) [17]. We have revealed that the heterozygous EK genotype has a high preventive effect against developing sCJD and V180I-gCJD [6,18].

PrP is negatively charged or neutral around the residue at position 219 in the wild type (WT), but the corresponding region is mostly positively charged in the E219K polymorphism [19,20]. At residue 200, the surface is negatively charged in WT, whereas it is positively charged in E200K, a similar point mutant that is pathogenic [20]. Dimerization of PrP specifically affects the structural dynamics of residues especially at the helix α1 (residues 143–157) or α3 (residues 199–224), which are essential for PrP^C^ to PrP^Sc^ conversion [21]. Among the residue-residue interactions in the dimer formation of the E200K mutant of PrP, the prominent electrostatic interaction of E219-K200 contributes to its stability [22]. Meanwhile, it remains unclear how electrostatic interactions affect PrP dimerization in 219 EK heterozygotes, and what molecular chemical mechanisms determine whether and to what extent they prevent the development of prion diseases (especially genetic prion diseases).

In this study, we aimed to update our previous reports on Japanese prion diseases [6,9,18,23], to conduct a replication effort with larger numbers of cases and statistically increased accuracy, and to evaluate the levels of effects of *PRNP* polymorphisms with odds ratios (ORs) on the onset of comprehensive prion diseases including sCJD, gCJD, dura-grafted iatrogenic CJD (dCJD), and Gerstmann-Straussler-Scheinker disease (GSS) using Japanese surveillance data on prion diseases. We showed that the surveillance study conducted in Japan for more than 20 years enabled us to compare the magnitude of the effect of heterozygosity on the development of prion diseases between the 129 MV and 219 EK genotypes, and to determine that the 219 EK heterozygosity has a preventive effect on disease onset not only in sCJD but also in genetic prion diseases. With the only exception in genetic prion diseases, the present study could not confirm a preventive effect of 219 EK heterozygosity on the development of E200K-gCJD. Hence, to understand such a mechanism of success or failure in preventing the pathogenesis by 219 EK heterozygosity, we proposed a hypothetical structural arrangement of its dimer formation, focusing in particular on the electrostatic interaction at residues 200 and 219 of PrP.

## 2 Results

### 2.1 Demographics of patients with major prion diseases and non-CJD patients

**Fig. 1:**
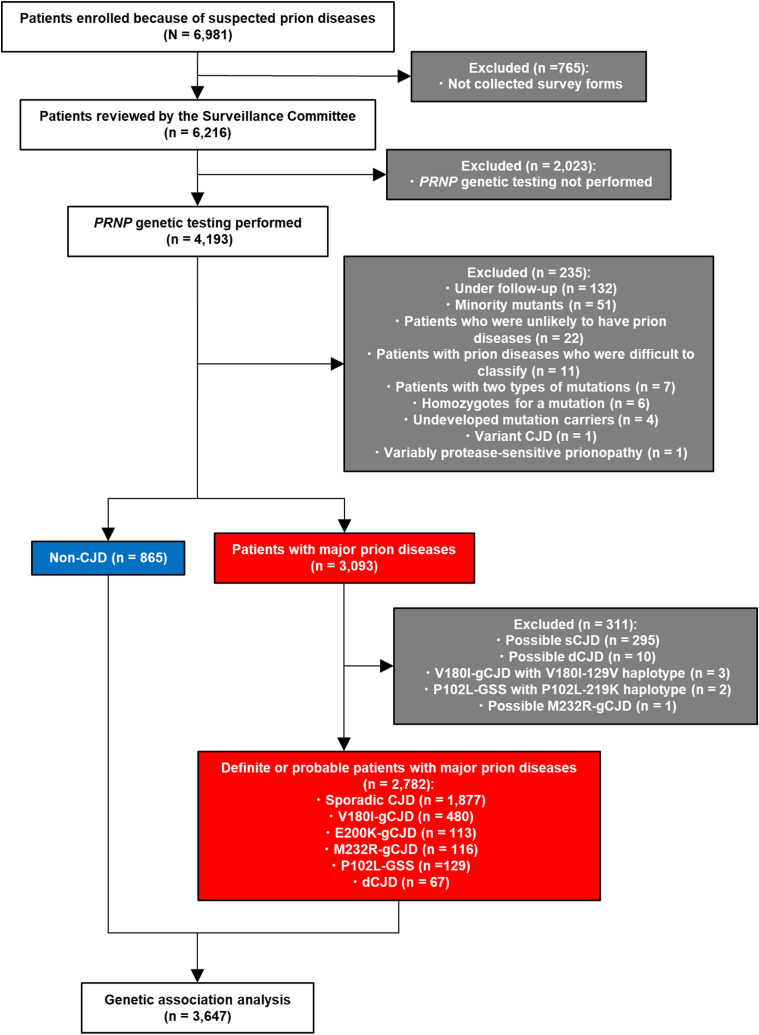
Selection procedure of patients for genetic association analysis of *PRNP* polymorphisms.

First, we summarized the profile of non-CJD controls and cases with prion diseases―sCJD, P102L-GSS, V180I-, E200K-, and M232R-gCJD, and dCJD (Table 1). Demographic data were based on survey forms collected by the Surveillance Committee in Japan. With respect to the study size, the number of patients with each type of prion disease and non-CJD was determined according to the flowchart procedure (Fig. 1).

**Table 1:** Demographics of patients with major prion diseases and non-CJD patients in Japanese CJD Surveillance.

	Sporadic CJD	Genetic CJD			GSS	dCJD	Non-CJD control
		V180I	E200K	M232R	P102L		
Total No.	1877	480	113	116	129	67	865
Sex							
Females, n (%)	1062 (56.58)	313 (65.21)	55 (48.67)	62 (53.45)	67 (51.94)	38 (56.72)	409 (47.28)
Males, n (%)	815 (43.42)	167 (34.79)	58 (51.33)	54 (46.55)	62 (48.06)	29 (43.28)	456 (52.72)
Median age at onset, y (IQR)^a^	70 (64–76)	79 (74–84)	62 (55–68)	67 (60–73)	57 (51.25–62)	61 (44–68)	–

See also Table S1. CJD, Creutzfeldt-Jakob disease; dCJD, dura-grafted iatrogenic CJD; GSS, Gerstmann-Straussler-Scheinker syndrome; IQR, interquartile range.

a On the age of onset, data were missing for sCJD (n = 4), V180I-gCJD (n = 4), and P102L-GSS (n = 1). P < 0.0001 (χ^2^ = 706.00) by Kruskal-Wallis test, and by Steel test difference in the onset age from the sporadic CJD cases with p < 0.0001 in genetic CJD with V180I or E200K mutation, GSS with P102L mutation, and dCJD, and with p = 0.0015 in genetic CJD with M232R mutation. Non-CJD is not listed because it is not a prion disease.

On the age of onset, data were missing for sCJD (n = 4), V180I-gCJD (n = 4), and P102L-GSS (n = 1). The age of onset was younger in E200K- (p < 0.0001), M232R-gCJD (p = 0.0020), P102L-GSS (p < 0.0001), and dCJD (p < 0.0001) than in sCJD, and older in V180I-gCJD (p < 0.0001) than in sCJD using nonparametric multiple comparisons (Steel test) with sCJD as control. No sex differences in age of onset were observed in these prion disease patients (Table S1).

### 2.2 129 MV heterozygosity affects onset of sCJD and V180I-gCJD

**Fig. 2:**
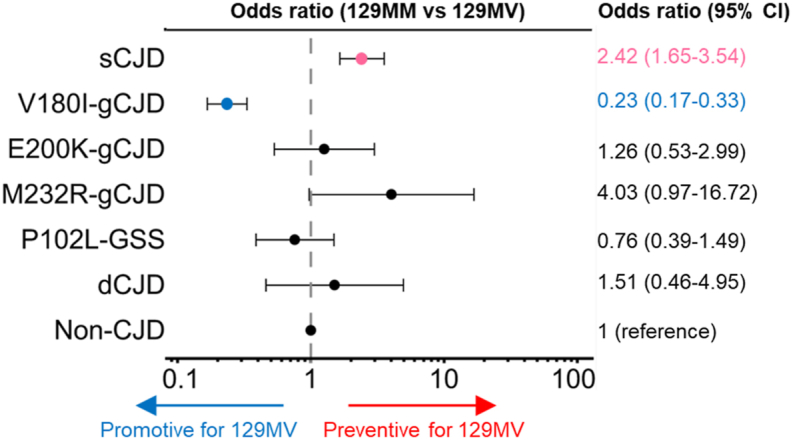
Effects of the polymorphic heterozygous genotype at codon 129 in *PRNP* on the onset of major Japanese prion diseases. Point estimates of OR and 95% CIs are shown as logarithmic axis in the forest plot. The dashed line (value of one) indicates the non-CJD control level as the reference. The p-values for the odds ratios for each prion disease were p < 0.0001 for sCJD, p < 0.0001 for V180I-gCJD, p = 0.50 for E200K-gCJD, p = 0.069 for M232R-gCJD, p = 0.50 for P102L-GSS, and p = 0.23 for dCJD.

To examine the effect of codon 129 polymorphism of *PRNP* on the disease onset, we analyzed genotypic data of prion diseases relative to non-CJD. We confirmed that the frequency of MM and MV genotypes was different between sCJD and non-CJD (p < 0.001; Table 2). The OR of MM for MV (OR_MM/MV_) was 2.42 (95% CI = 1.65–3.54, p < 0.0001), indicating that heterozygosity has a low preventive effect on the development of sCJD (Fig. 2).

**Table 2:** Frequency of *PRNP* polymorphic genotypes in Japanese major prion diseases and the non-CJD control.

	Codon 129, n (%)					Codon 219, n (%)				
	MM	MV	VV	χ^2^	p	EE	EK	KK	χ^2^	p
sCJD	1813 (96.59)	53 (2.82)	11 (0.59)	21.56	<0.001	1865 (99.52)	8 (0.43)	1 (0.05)	153.52	<0.001
V180I-gCJD	369 (76.88)	111 (23.13)	0 (0)	77.07	<0.001	477 (100)	0 (0)	0 (0)	48.45	<0.001
E200K-gCJD	107 (94.69)	6 (5.31)	0 (0)	0.28	0.60	107 (94.69)	6 (5.31)	0 (0)	2.18	0.14
M232R-gCJD	114 (98.28)	2 (1.72)	0 (0)	4.29	0.038	115 (99.14)	1 (0.86)	0 (0)	9.88	0.002
P102L-GSS	118 (91.47)	11 (8.53)	0 (0)	0.66	0.42	113 (97.41)	3 (2.59)	0 (0)	6.21	0.013
Dura-grafted iCJD	64 (95.52)	3 (4.48)	0 (0)	0.46	0.50	60 (92.31)	5 (7.69)	0 (0)	0.24	0.62
Non-CJD control	807 (93.29)	57 (6.60)	1 (0.12)	Reference	–	777 (90.24)	82 (9.52)	2 (0.23)	Reference	–

See also Tables S2 and S3. CJD, Creutzfeldt-Jakob disease; gCJD, genetic CJD; GSS, Gerstmann-Straussler-Scheinker syndrome; iCJD, iatrogenic CJD; sCJD, sporadic CJD. Pearson's χ^2^ test was performed for MM and MV genotypes at codon 129 and for EE and EK genotypes at codon 219 compared to non-CJD.

We also evaluated the effects of genotypes at codon 129 on the onset of gCJD, GSS, and dCJD. We found that the frequency of MM and MV genotypes in V180I-gCJD was clearly distinct from that in non-CJD (p < 0.0001; Table 2). OR_MM/MV_ was 0.23 (95% CI = 0.17–0.33, p < 0.0001; Fig. 2) and its heterozygosity had a moderate promotive effect (1/OR_MM/MV_ = 4.26) on the onset of V180I-gCJD. Regarding the age of onset, patients with the MV genotype showed an earlier onset of disease than those with the MM genotype (p = 0.024, Wilcoxon rank-sum test; Table S2). The frequencies of MM and MV genotypes in E200K-gCJD (p = 0.60), P102L-GSS (p = 0.42), and dCJD (p = 0.50) were not apparently diverse from that in non-CJD. Meanwhile, these frequencies were slightly different in M232R-gCJD than in non-CJD (p = 0.038). The ORs_MM/MV_ were p > 0.05 in E200K-gCJD (OR_MM/MV_ = 1.26, 95% CI = 0.53–2.99, p = 0.50), M232R-gCJD (OR_MM/MV_ = 4.03, 95% CI = 0.97–16.72, p = 0.069), P102L-GSS (OR_MM/MV_ = 0.76, 95% CI = 0.39–1.49, p = 0.50) and dCJD (OR_MM/MV_ = 1.51, 95% CI = 0.46–4.95, p = 0.23).

Here, no adjustment for sex or age of onset was done in the calculation of ORs_MM/MV_. There was no sex difference by the genotype of codon 129 in patients with all types of prion diseases and non-CJD patients (Table S3).

### 2.3 219 EK heterozygosity prevents the development of broad-spectrum prion diseases

**Fig. 3:**
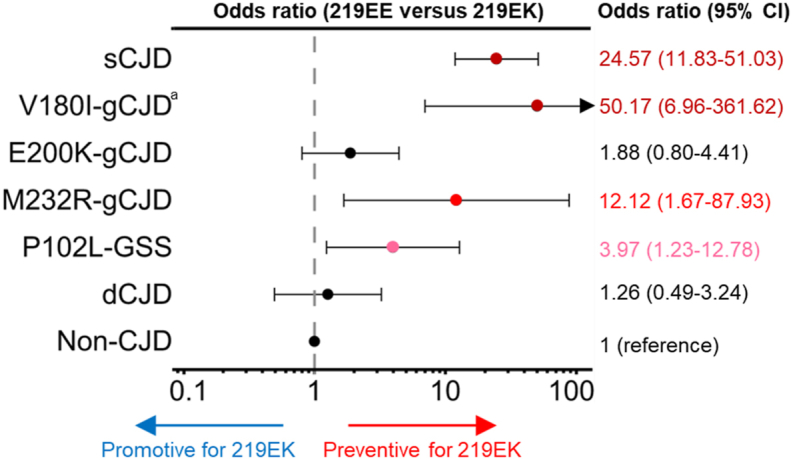
Effects of polymorphic heterozygous genotypes at codon 219 in *PRNP* on the onset of major Japanese prion diseases. Point estimates of OR and 95% CI are shown on the logarithmic axis. The dashed line (value of one) indicates the non-CJD control level as the reference. The p-values for the ORs for each prion disease were p < 0.0001 for sCJD, p = 0.0002 for V180I-gCJD, p = 0.148 for E200K-gCJD, p = 0.020 for M232R-gCJD, p = 0.023 for P102L-GSS, and p = 0.66 for dCJD. ^a^The hypothetical odds ratio and the 95% CI for V180I-gCJD were calculated by the supposition that one patient with the 219 EK genotype was included in our surveillance data.

We analyzed the relationship between the polymorphism of *PRNP* at codon 219 and the prion disease onset by comparing it with non-CJD. The frequency of EE and EK genotypes in sCJD varied from that in non-CJD (p < 0.001; Table 2). The OR of EE versus EK (OR_EE/EK_) in sCJD was 24.57 (95% CI = 11.83–51.03, p < 0.0001) when non-CJD was used as a reference (Fig. 3). This result confirmed that the EK genotype has a highly preventive effect on the development of sCJD. Conversely, the frequencies of these genotypes were not different in dCJD from non-CJD (p = 0.62), with the OR_EE/EK_ being 1.26 (95% CI = 11.83–51.03, p = 0.66).

In genetic prion diseases, the differences of EE and EK genotypic proportions in V180I-gCJD (p < 0.001), M232R-gCJD (p = 0.002), and P102L-GSS (p = 0.013) were established as compared with non-CJD (Table 2). No V180I-gCJD case with 219 EK was observed, suggesting that the EK heterozygosity highly protects against the V180I-gCJD onset. Even assuming there was one V180I-gCJD patient with the 219 EK genotype for comparison of the OR_EE/EK_ with the other prion diseases, this heterozygosity had a high preventive effect on disease development (OR_EE/EK_ = 50.17, 95% CI = 6.96–361.62, p = 0.0002; Fig. 3). Furthermore, as for other genetic prion diseases, ORs_EE/EK_ indicated that 219 EK heterozygosity had a low preventive effect for P102L-GSS (OR_EE/EK_ = 3.97, 95% CI = 1.23–12.78, p = 0.023), and a moderate preventive effect for M232R-gCJD (OR_EE/EK_ = 12.12, 95% CI = 1.67–87.93, p = 0.020). Exceptionally, neither the rate of EE and EK genotypes nor the OR_EE/EK_ (OR_EE/EK_ = 1.88, 95% CI = 0.80–4.41) were p > 0.05 for E200K-gCJD compared to non-CJD.

As in codon 129, the effects of sex and age at onset were not controlled for in the computation of ORs_EE/EK_. Similar to codon 129, codon 219 also showed no evident sex differences between genotypes in patients with each type of prion disease and non-CJD patients (Table S3). Concerning the age of onset, the number of patients with the EK genotype was so small compared to those with the EE genotype that it was difficult to statistically assess the difference (Table S2).

## 3 Discussion

This study is a *PRNP* genetic analysis available based on the surveillance of prion diseases in Japan. We analyzed ∼1900 cases of sCJD, more than 100 cases of each genetic prion disease, and 67 cases of dCJD, which we were able to analyze by continuing the prion disease surveillance for about 20 years. We aimed to show and compare the effect of heterozygosity on the normal polymorphisms at codons 129 and 219 that resulted in the prevention or promotion of comprehensive prion diseases using ORs. A long-term surveillance study in Japan demonstrated that heterozygotes of the 219 EK genotype can be evaluated for their efficacy in preventing the onset of not only sCJD but also genetic prion diseases.

In sCJD, the 129 MV heterozygosity showed a low preventive effect (OR_MM/MV_ = 2.42) on the onset of the disease in Japan. This result is consistent with previous reports that the 129 MM genotype predisposes to sCJD, based on previous studies mainly in Western countries and recent data from a prion disease surveillance survey also in Japan [13,[23], [24], [25], [26], [27], [28]]. Since the 129 MM and 129 VV genotypes are overrepresented in sCJD, while the 129 MV genotype is underrepresented, conformational conversion from PrP^C^ to PrP^Sc^ has been considered more efficient in individuals homozygous for codon 129 than in those heterozygous for this codon [15,16,25,[29], [30], [31]].

As for genetic prion diseases, the 129 MM genotype is predisposed to M232R-gCJD according to another Japanese prion disease surveillance survey recently [23]. Probably because of differences in not performing multivariate analysis and furthermore not combining non-CJD patients with 129 MV and 129 VV genotypes into one group when analyzing logistic regression, our analysis did not confirm a preventive effect of 129 MV heterozygosity on the development of any of the genetic prion diseases. Meanwhile, the percentages of 129 MV and 129 VV genotypes in E200K-gCJD are much lower than in normal individuals in European countries [32,33]. The discrepancy may have been created by the small number of E200K-gCJD patients with the 129 MV genotype in Japan due to the different general frequency of the 129 V allele. Oppositely, we confirmed that the 129 MV heterozygosity has a moderate promotive effect on the development of V180I-gCJD (1/OR_MM/MV_ = 4.26), as previously reported [6,23]. Currently, it is still uncertain whether wild-type PrP^C^ with the V129 residue promotes de novo PrP^Sc^ synthesis derived from the V180I-129M haplotype or accelerates the V180I mutant PrP^Sc^ propagation.

In codon 219, the heterozygosity exhibited a high preventive effect on developing sCJD overall (OR_EE/EK_ = 24.57). Until now, the relationship between heterozygosity at codon 219 and the development of genetic prion diseases and the levels of its effects have not been fully investigated. In the present study, the 219 EK heterozygosity had a significant preventive effect on the disease onset at high (OR_EE/EK_ > 50.17) and moderate (OR_EE/EK_ = 12.12) levels for gCJD with low penetrance mutations of V180I (∼1% lifetime risk) and M232R (∼0.1% lifetime risk), respectively, and at a low level (OR_EE/EK_ = 3.97 for P102L-GSS) for a genetic prion disease with a high penetrance mutation of P102L (100% estimated penetrance) [34]. Interestingly, in the conflict between the promotive genetic prion diseases and the preventive polymorphism, no heterozygous inhibition by 219 EK was observed at all in E200K-gCJD with incomplete penetrance (⁓40%) [35].

**Fig. 4:**
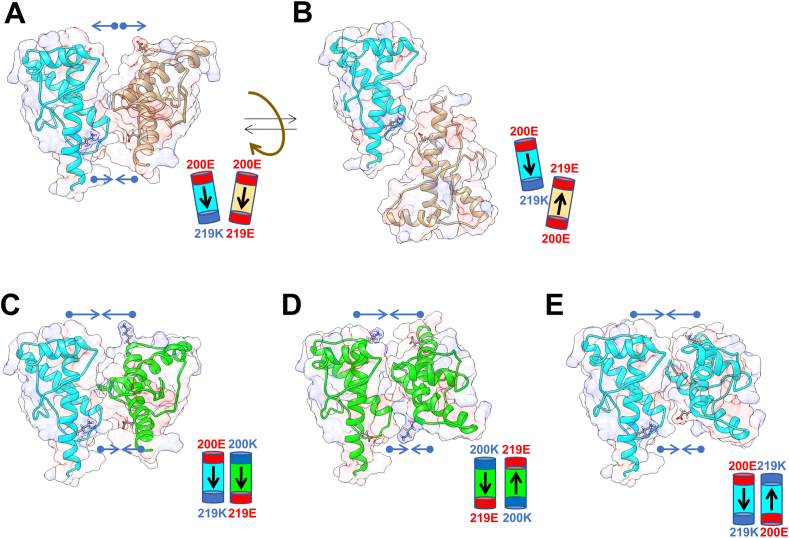
Mutant PrP homodimer formation by electrostatic interaction defeats the effect of 219 EK heterozygosity in preventing pathogenesis in E200K-gCJD. Structural image of human PrP^C^ α3-α3 dimer formation Structural data from the Protein Data Bank (ID: 6DU9) [21] was visualized using the Chimera program [40] as a ribbon model. Electrostatic surface pattern was generated by the Chimera APBS tool [41]. The ribbon model of PrP mutants, (200E, 219K), (200K, 219E), and (200E, 219E), are shown as cyan, green, and brown, respectively. (A and B) (200E, 219K) - (200E, 219E) dimer model. (C) (200E, 219K) - (200K, 219E) dimer model. (D) (200K, 219E)_2_ dimer model. (E) (200E, 219K)_2_ dimer model.

It has been proposed that the monomer-dimer equilibrium of PrP^C^ plays a critical role in the molecular-level processes involved in prion [[36], [37], [38]]. Recent recombinant PrP crystallographic analyses focusing on packing have revealed that diverse patterns in α3-α3 interactions stabilized by multiple electrostatic interactions, hydrogen bonds, and hydrophobic interactions can be formed in various relative molecular orientations [21]. The aforementioned facts suggest that electrostatic interactions may be involved in the protective mechanism. This electrostatic interaction increases the fraction of 219E and 219K molecules forming a heterodimer between them and is consequently protective (Fig. 4A and B). On the other hand, the presence of the E200K mutation on the 219 E allele makes it possible to form heterodimer as in Fig. 4C, homodimer composed of mutants as in Fig. 4D, and homodimer composed of wild type only as in Fig. 4E. In brief, these homodimers will be feasible to be converted to the abnormal PrP molecules. In contrast, P102L, V180I, and M232R, which are frequently observed in Japan, are amino acid mutations that do not affect the ionic charge and are not located on the α-helix 3. Thus, under these situations, E219K can form the preventive heterodimer through the electrostatic interaction. In the case of 129 MV heterozygote, such protective heterodimer formation by electrostatic interactions would not be predominant. Thus, a mixture of homodimer and heterodimer may make it difficult to counteract the promotive gCJD mutations. Such modulations of dimer via electrostatic interactions can be adapted to the conspicuous difference in the preventive effect in sCJD. Breifly, 219 EK (OR_EE/EK_ = 24.57) in sCJD would form the preventive heterodimer dominantly, on the other hand, 129 MV (OR_MM/MV_ = 2.42) in sCJD would form dimers feasible to be converted. The control of the heterodimer formation by electrostatic interactions is expected to be developed as a novel prophylactic for CJD.

Unfortunately, in Japan, we have more patients who received PrP^Sc^-contaminated dural grafts than in the other countries [39]. Therefore, the larger number of dCJD cases enabled us to analyze both codon 129 and 219 polymorphisms of *PRNP* in the current study. As opposed to sCJD, the heterozygosity in both 129 MV and 219 EK did not prevent the onset of dCJD. The outstanding finding was the marked difference in ORs_EE/EK_ at codon 219 between sCJD and dCJD. These results indicate that the preventive effect of heterozygosity at codon 219 is limited to de novo synthesis of PrP^Sc^ (initial formation of PrP^Sc^) in sCJD or most of genetic prion diseases and the heterozygosity has no inhibitory effect on the acceleration or amplification of existing PrP^Sc^ in dCJD. This was in agreement with a report that the 219 EK genotype was infected with the M1 prion strain in an inoculation experiment on knock-in mice carrying human *PRNP* [6]. This means that experimental transmission studies may be useful for predicting the onset of acquired prion diseases but not sCJD and genetic prion diseases.

This study has a limitation in that it did not consider the effect of genetic relatedness when assessing the effect of *PRNP* polymorphisms in the development of genetic prion diseases. Especially when assessing the OR for the effect of the codon 219 polymorphism on the development of P102L-GSS, we cannot rule out the possibility that it is more strongly attributable to the 219 K allele frequency in larger families, given the near 100% penetrance of the P102L mutation [34]. For the same reason, the effect of genetic relatedness on OR_EE/EK_ may not be eliminated even for the E200K mutation, which has a relatively high penetrance (40%) [35]. The lower limits of the 95% CI for ORs_EE/EK_ in E200K-gCJD and P102L-GSS were 0.80 and 1.23, respectively, close to 1. In addition, because the number of patients with most prion diseases is not large, and the number of heterozygotes among them is small, the 95% CI ranges of these ORs are wide, and the exact effect of heterozygosity could not be fully assessed. It cannot be ruled out that some of the analyses in this study may have statistical power problems due to insufficient numbers of cases. Therefore, a larger surveillance study including East Asian countries with high 219 K allele frequencies may be needed to assess whether the 219 EK genotype is bona fide preventive against the development of these genetic prion diseases. As described in a previous similar study, limitations of prion disease surveillance studies in Japan include the low proportion of autopsy-confirmed prion disease patients and the fact that control patients are not healthy subjects [23]. If each prion disease is analyzed in terms of subtypes, there may be differences in the effect of heterozygosity at codon 129 or codon 219 on disease onset, but this was not addressed in this study. To address concerns about the limitations of the current study, the effect of 129 MV or 219 EK heterozygosity on prion disease pathogenicity needs to be tested in an experimental system using cloned cells with the mutations.

Concerning generalizability, the results obtained in this study can be easily applied to East Asian ethnic groups with a particularly high genetic background similarity to the Japanese and may be applicable to a wider range of ethnic groups given the high validity of the results due to the size of the number of patients evaluated. The pathogenicity of various prion mutations is controversial. Since the propagation of PrP^Sc^ would be caused by the internal domain and external prion binding dynamics, our hypothesis and study may provide insight to other researchers. Furthermore, the present findings on the effect of heterozygosity for polymorphisms of *PRNP* on the development of comprehensive prion diseases are not only applicable to epidemiological or genetic facts but are also useful in the molecular biological or structural chemical interpretation of the mechanisms of pathogenesis.

## Star methods

### Experimental model and subject details

#### Study design and patients

The data on the totality of patients in this study were collected nationwide in Japan by the Creutzfeldt-Jakob disease Surveillance Committee between April 1999, and February 2022, as previously reported [9]. Neurologists throughout Japan requested that the *PRNP* gene and cerebrospinal fluid be tested by specialized institutions on patients suspected of having a prion disease. These patients were diagnosed with prion diseases or as non-CJD patients who were once suspected to have a prion disease only to be found prion disease-free by the follow-up according to the Masters and/or WHO criteria [42,43]. Since there have been no reports to date showing an association between the *PRNP* gene and neurological diseases other than prion disease [44,45], these non-CJD patients were established as the control group in the genetic association analysis. Almost all patients analyzed were Japanese. A few patients were of non-Japanese East Asian origin, but the frequencies of *PRNP* polymorphisms were almost ethnically identical [46]. Demographic data were based on survey forms collected by the Surveillance Committee. With respect to the study size, the number of patients with each type of prion disease and non-CJD was determined according to the flowchart procedure (Fig. 1). No matching between prion disease patients and non-CJD patients was performed.

In the present study, patients who were used for analyses in previous reports were also included [6,9,23,47]. In prion diseases, the patients analyzed in this study were either definite or probable cases of sCJD, gCJD (V180I, E200K, M232R), GSS (P102L), or dCJD. To precisely assess the implication of each *PRNP* polymorphism for prion diseases, we ruled out prion disease patients harboring a pathogenic mutation with the 129 V or 219 K haplotype, and gCJD patients with the homozygous variants (E200K or M232R), or with two different mutations (V180I and M232R) on the respective alleles.

The use of information about the patients and samples from them was approved by the institutional review boards in each institution. Written informed consent for the analyses of clinical data and samples and the autopsies was provided by all the patients or their next of kin.

### Method details

#### Genetic analysis

DNA from patients was extracted from peripheral blood ante mortem and/or frozen brains after death. After extraction, DNA was stored in TE buffer at 4 °C. The genotypes of *PRNP* polymorphism at codon 129 and 219, and the presence of mutations were identified by DNA sequencing and restriction fragment length polymorphism analyses at the Department of Neurological Sciences, Tohoku University Graduate School of Medicine as reported previously [48]. DNA sequencing was performed using the BigDye Terminator v3.1 Cycle Sequencing Kit (Applied Biosystems) with the module set to BDx_LongSeq80_POP7 on 3130×/Genetic Analyzers (Applied Biosystems). DNA sequencing data were analyzed using DNA Sequencing Analysis Version 5.3.1 software (Applied Biosystems). The accuracy of the basecalling was 98.5%, with an error rate of less than 2%. The allele analysis was performed by cloning the PCR products [49]. Genetic testing was performed in small batches sequentially after receiving patient blood from neurologists throughout Japan.

### Quantification and statistical analysis

#### Hardy-Weinberg equilibrium

Hardy-Weinberg equilibrium was considered for the purpose of examining whether the non-CJD patient group is comparable to the general population in the genetic association analysis of *PRNP* polymorphisms. In non-CJD, M and V allele frequencies at codon 129 were 0.966 and 0.034, respectively. Fisher's exact test for the genotype frequency evaluated the expected values based on allele frequencies and the number of patients (p = 1.00), confirming complete Hardy-Weinberg equilibrium. The allele frequencies at codon 219 were 0.950 for E and 0.050 for K, confirming complete Hardy-Weinberg equilibrium as well (p = 1.00). On the other hand, sCJD had more patients available for the analysis than non-CJD, but the M and V allele frequencies at codon 129 were in disequilibrium at 0.980 and 0.020, respectively (p = 0.002; Fisher's exact test). The allele frequencies of codon 219 in sCJD were 0.997 for E and 0.003 for K, in Hardy-Weinberg equilibrium (p = 0.65; Fisher's exact test). Based on these results, we concluded that the distributions of genotypes at codons 129 and 219 were in complete Hardy-Weinberg equilibrium in non-CJD patients, unlike those in sCJD, and therefore could be treated as equivalent to the general population to some extent.

#### Statistical analysis for quantitative variables

For the goodness of fit in the ages at onset, the normality was assessed using Shapiro-Wilk test for each group or subgroup. The age in each patient group except for E200K-gCJD was non-normally distributed (p < 0.01). We analyzed the ages at onset with Kruskal-Wallis test (analysis of variance for nonparametric data) to examine the equality of the medians across groups and Steel test was performed to control for the risk of false positive findings when conducting multiple comparisons against the sCJD group. However, differences in age at onset between males and females in each prion disease (Table S1) or between 129 MM and 129 MV genotypes in V180I-gCJD (Table S2) were analyzed by Wilcoxon rank-sum test. The test calculated the statistic S, which is the sum of the rank scores for the level with the smaller number of observations. Except for V180I-gCJD with 129 MM and 129 MV, we did not perform statistical tests to evaluate differences in age at onset because the number of patients with MV and VV for MM at codon 129 and EK and KK for EE at codon 219 in each prion disease is very small (Table S2).

#### Contingency analysis

We analyzed the distribution in categorical variables using Pearson's χ^2^ test or Fisher's exact test that was for small sample sizes or sparse tables. The p-values in Fisher's exact test were bilateral. For Fisher's exact test, the probability P of the observed table occurring was also calculated along with the calculated p-value. Even among patients with sCJD and non-CJD, the number of the 129 VV and 219 KK genotypes was very small. Therefore, we considered it difficult to evaluate the effect of these genotypes on the development of prion disease in comparison with non-CJD patients and limited the contingency analysis to the MM and MV genotypes for codon 129 and the EE and EK genotypes for codon 219 (Table 2). However, all genotypes were included when analyzing whether there were differences in the distribution of genotypes at codon 129 or 219 between males and females in each prion disease and non-CJD patients (Table S3).

#### Logistic regression analysis

We analyzed ORs and Wald-based 95% CIs by logistic regression. Since bias resulting from genotypes (129 VV and 219 KK) and haplotypes (P102L-219K and V180I–129V), which were present in only a few cases, could not be ignored, we excluded cases with them when performing logistic regression analysis. Additionally, Non-CJD patients included a variety of neurological diseases and were heterogeneous in age of onset. Therefore, no adjustment by age of onset was made to exclude the influence of bias when obtaining ORs.

We compared crude OR values calculated with reference to genotypes of non-CJD controls for codon 129 or 219 of *PRNP* to determine how preventive or vulnerable heterozygotes are versus homozygotes (129 MM or 219 EE: exposure) with prion disease development as an outcome in each prion disease. For simplicity of expression, the degree of the preventive effect among heterozygotes for which OR was observed to be significant (p < 0.05) was defined as low (OR < 4), moderate (4 ≤ OR < 13), and high (OR ≥ 13), and the reciprocal of OR was applied to this definition for the degree of the promotion effect.

#### General statistical analysis

In each statistical analysis, where relevant data were missing, the corresponding patients were excluded from the analysis. Patients classified as being under follow-up included those who were not available for follow-up. Such patients were excluded from the genetic association analysis. The analysis in this study did not consider the influence of genetic relatedness. All the statistical analyses were performed using JMP Pro 16.2.0 (SAS Institute Inc.).

## Author contribution statement

Hideaki Kai, Kenta Teruya, Tetsuyuki Kitamoto: Conceived and designed the experiments; Performed the experiments; Analyzed and interpreted the data; Contributed reagents, materials, analysis tools or data; Wrote the paper.

Atsuko Takeuchi: Performed the experiments; Contributed reagents, materials, analysis tools or data.

Yoshikazu Nakamura, Hidehiro Mizusawa, Masahito Yamada: Contributed reagents, materials, analysis tools or data.

### Data availability statement

Data will be made available on request.

## Declaration of interest's statement

The authors declare no competing interests.
