# Supplementary material for: Preventive or promotive effects of PRNP polymorphic heterozygosity on the onset of prion disease
Source: Heliyon. 2023 Feb 24;9(3):e13974. doi: 10.1016/j.heliyon.2023.e13974 (PMC10006469; doi:10.1016/j.heliyon.2023.e13974)
Supplement: Multimedia component 1 [file mmc1.pdf]

Table S1. Age of onset and its comparison by sex for each prion disease, related to Table 1

	Sporadic CJD	Genetic CJD			GSS	dCJD
		V180I	E200K	M232R	P102L	
**Females**						
**n**	1,058	310	55	62	67	38
**Median age at onset, y (IQR)**	70 (64-76)	79 (75-85)	61 (55-68)	66 (60-74.25)	56 (46-62)	62.5 (47-68)
**Males**						
**n**	815	166	58	54	61	29
**Median age at onset, y (IQR)**	70 (64-76)	79 (73-83.25)	63 (54.75-68)	68.5 (62.75-73)	58 (53.5-63)	59 (43-69)
**Wilcoxon test**						
**S**	759,430	37,018.5	3,080	3,347	4,256.5	997
**p-value**	0.72	0.072	0.75	0.30	0.12	0.89

CJD, Creutzfeldt-Jakob disease; dCJD, dura-grafted iatrogenic CJD; GSS, Gerstmann-Straussler-Scheinker syndrome; IQR, interquartile range. Assessment of normality of age at onset by the Shapiro-Wilk test showed $p > 0.05$ for females with E200K-gCJD ($p = 0.69$), females with M232R-gCJD ($p = 0.092$), females with P102L-GSS ($p = 0.20$) and males with dCJD ($p = 0.077$). Comparison of males and females in each prion disease was performed by Wilcoxon rank-sum test for nonparametric analysis.

Table S2. Age at onset of prion disease for each *PRNP* genotype at codon 129 and 219, related to Table 2

	Codon 129			Codon 219		
	**MM**	**MV**	**VV**	**EE**	**EK**	**KK**
**sCJD**						
**n**	1,809	53	11	1,861	8	1
**Median, y (IQR)**	70 (64-76)	68 (61-73.5)	74 (77-66)	70 (64-76)	70.5 (59.5-78)	58
**V180I**	S = 23147.5, p = 0.024[a]					
**n**	367	109	0	473	0	0
**Median, y (IQR)**	79 (75-85)	78 (72-82)	NA	79 (74-84)	NA	NA
**E200K**						
**n**	107	6	0	107	6	0
**Median, y (IQR)**	63 (68-55)	60 (53-68.75)	NA	62 (54-67)	67.5 (64.75-76.5)	NA
**M232R**						
**n**	114	2	0	115	1	0
**Median, y (IQR)**	67 (60–73)	64 (57–71)	NA	67 (60-73)	65	NA
**P102L**						
**n**	117	11	0	112	3	0
**Median, y (IQR)**	57 (51–62)	57 (54–61)	NA	56.5 (50.25-61)	56 (46-68)	NA
**dCJD**						
**n**	64	3	0	60	5	0
**Median, y (IQR)**	60（42.5-68）	68 (57-70)	NA	61.5 (45.25-68)	67 (43.5-76.5)	NA

sCJD = Sporadic Creutzfeldt-Jakob disease; dCJD = dura-grafted iatrogenic CJD; NA = not available. Except for V180I-gCJD with 129MM and 129MV, we did not perform statistical tests to evaluate differences in age at onset because the number of patients with MV and VV for MM at codon 129 and EK

and KK for EE at codon 219 in each prion disease is very small. Note: Regarding the genetic analysis of *PRNP*, in some cases, only the genotype at codon 129 was analyzed and the genotype at codon 219 was not available. [a]Wilcoxon rank-sum test was performed.

Table S3. Percentage of females and males per *PRNP* genotype at codon 129 and 219, related to Table 2

	Codon 129			Codon 219		
	**MM**	**MV**	**VV**	**EE**	**EK**	**KK**
**sCJD**	$\chi^2 = 0.88$, p＝0.64			P = 0.15, p = 1.00		
**Females, n (%)**	1,029 (56.76)	28 (52.83)	5 (45.45)	1,054 (56.51)	5 (62.50)	1 (100)
**Males, n (%)**	784 (43.24)	25 (47.17)	6 (54.55)	811 (43.49)	3 (37.5)	0 (0)
**V180I**	$\chi^2 = 2.81$, p＝0.093			P = 1, p = 1.00		
**Females, n (%)**	248 (67.21)	65 (58.56)	0 (NA)	310 (64.99)	0 (NA)	0 (NA)
**Males, n (%)**	121 (32.79)	46 (41.44)	0 (NA)	167 (35.01)	0 (NA)	0 (NA)
**E200K**	$\chi^2 = 0.004$, p＝0.95			$\chi^2 = 0.82$, p＝0.36		
**Females, n (%)**	52 (48.60)	3 (50)	0 (NA)	51 (47.66)	4 (66.67)	0 (NA)
**Males, n (%)**	55（51.40）	3 (50)	0 (NA)	56 (52.34)	2 (33.33)	0 (NA)
**M232R**	$\chi^2 = 0.010$, p＝0.92			$\chi^2 = 0.82$, p＝0.36		
**Females, n (%)**	61 (53.51)	1 (50)	0 (NA)	62 (53.91)	0 (0)	0 (NA)
**Males, n (%)**	53 (46.49)	1 (50)	0 (NA)	53 (46.09)	1 (100)	0 (NA)
**P102L**	$\chi^2 = 1.169$, *p*＝0.28			$\chi^2 = 0.38$, p＝0.54		
**Females, n (%)**	63 (53.39)	4 (36.36)	0 (NA)	55 (48.67)	2 (66.67)	0 (NA)
**Males, n (%)**	55 (46.61)	7 (63.64)	0 (NA)	58 (51.33)	1 (33.33)	0 (NA)
**dCJD**	$\chi^2 = 2.40$, p = 0.12			$\chi^2 = 3.30$, p = 0.07		
**Females, n (%)**	35 (54.69)	3 (100)	0 (NA)	37 (61.67)	1 (20)	0 (NA)

**Males, n (%)**	29（45.31）	0 (NA)	0 (NA)	23 (38.33)	4 (80)	0 (NA)
**Non-CJD**	P = 0.049, p = 0.68			P = 0.017, p = 0.31		
**Females, n (%)**	380 (47.09)	29 (50.88)	0 (NA)	370 (47.56)	36 (43.9)	2 (100)
**Males, n (%)**	427 (52.91)	28 (49.12)	1 (100)	408 (52.44)	46 (56.1)	0 (0)

sCJD = Sporadic Creutzfeldt-Jakob disease; dCJD = dura-grafted iatrogenic CJD; NA = not available. The contingency tables were tested by Pearson's χ^2 test or Fisher's exact test, as appropriate. For the Fisher's exact test, the probability P of the observed table occurring and the p-value calculated by the test are shown. Note: Regarding the genetic analysis of *PRNP*, in some cases, only the genotype at codon 129 was analyzed and the genotype at codon 219 was not available. In addition to the cases in Table S2, patients with unknown age of onset are included.
